# Idiopathic chilblain: a case series from Injibara General Hospital, Ethiopia

**DOI:** 10.3389/fmed.2025.1564283

**Published:** 2025-03-18

**Authors:** Alemu Bezabih Tegegnie, Tamiru Alene, Meaza Molla Sineshaw

**Affiliations:** ^1^Department of Dermatovenereology, College of Medicine and Health Sciences, Injibara University, Injibara, Ethiopia; ^2^Department of Pediatrics and Child Health Nursing, College of Medicine and Health Sciences, Injibara University, Injibara, Ethiopia; ^3^Department of Public Health, College of Medicine and Health Sciences, Injibara University, Injibara, Ethiopia

**Keywords:** pernio, chilblain, case series, dermatology, Injibara

## Abstract

**Background:**

Idiopathic chilblains are inflammatory lesions on the skin that typically appear after being in a cold, wet environment. The condition manifests itself as burning, soreness, and pruritis in the extremities (fingers and toes), usually occurring 12 to 24 h following a triggering event. Conservative measures are the mainstay of treatment for idiopathic chilblain; however pharmaceutical therapy may be necessary in cases that are severe or persistent. This is the only case series in Ethiopia concerning idiopathic chilblain.

**Case presentation:**

From June to August of 2024, 12 individuals in the dermatology department of Injibara General Hospital in Ethiopia were diagnosed with idiopathic chilblain. Nine (or 75%) of these patients were women. This study’s mean age at diagnosis was 23.2 years (range: 3–55 years). All cases presented with itching sensations and/or pain. Only a few patients presented with swelling and color change during cold exposure. One patient presented with ulceration involving the tip of the digits. Most patients were prescribed clobetasol propionat and oral nifedipine. Others were treated with only topical corticosteroids, and few refused any medical treatment. All patients were counseled to avoid cold and keep extremities warm. Only one patient has persistent disease and the rest have completely improved.

**Conclusion:**

According to the current study, young women were the group most frequently impacted by idiopathic chilblain. Idiopathic chilblain in Ethiopia is underreported, necessitating comprehensive studies involving larger patient numbers and a focus on prevalence, diagnosis, treatment, and psychosocial impact.

## Introduction

Idiopathic chilblains are inflammatory lesions on the skin that typically appear after being in a cold, wet environment (1). When chilblain lesions arise without immune-mediated inflammatory disease, they are referred to more broadly as perniosis (2). Although it is seen everywhere, persons residing in colder regions like the United Kingdom and Northwestern Europe are at higher risk of developing idiopathic chilblains (3). However, more recent studies are reporting increasing numbers of cases from other parts of the world, possibly due to climate changes.

The exact pathogenesis of idiopathic chilblains is unknown. Several studies have described the process as a vasculopathy. It is hypothesized that susceptible individuals may be at risk of developing these lesions secondary to a disruption of normal neurovascular responses to dermal temperature changes (4). The concept of a cold-induced vasodilatory reflex has been described as a protective physiologic mechanism that intermittently opens blood flow to allow reperfusion and prevent skin ischemia (5). Studies have shown abnormally high levels of vasoconstrictive agents such as endothelin-1 in subjects with idiopathic Raynaud phenomenon and acrocynosis (6). It has been suggested that patients with chilblains have persistent or prolonged cold-induced vasoconstriction leading to hypoxemia and a subsequent secondary inflammatory reaction (7–10).

Chilblains are almost always seen during the months of November to April under nonfreezing and damp conditions. It is thought that humidity adds to the chilling effect by enhancing thermal conductivity (11). The characteristic lesions usually appear within a few hours of cold exposure of the affected area (7, 8). Although chilblains occur in all ages and sexes, its female predominance might reflect choices of footgear (4). The condition manifests itself as burning, soreness, and pruritis in the extremities (fingers and toes), usually occurring 12 to 24 h following a triggering event (1). The lesions are usually pruritic at first, then turning tender. They can be erythematous to violaceous macules, tender papules, plaques, or nodules (which can develop central erosions/ulceration) (12). Symptoms begin to occur in the winter, with individual bouts lasting 1–3 weeks and spontaneous remission in the spring. Annual recurrences are typical, and a long course may be experienced by elderly patients (13).

Conservative measures are the mainstay of treatment for idiopathic chilblain; however, pharmaceutical therapy may be necessary in cases that are severe or persistent (13). One of the cornerstones of systemic treatment is nifedipine (3). Corticosteroids are another treatment with little evidence (3). Without an objective control, it is challenging to evaluate the effectiveness of idiopathic chilblain therapy given the spontaneous remissions. We report the first case series of idiopathic chilblains from Ethiopian patients.

## Methods

Ethiopia has three different seasons: the Belg (February to May), the Kiremt (June to September), and the Bega (October to January). It’s main wet season and the coldest is Kiremt. The highest recorded temperature in Injibara city was 31.22° Celsius in April, while the lowest recorded temperature was 7.44° Celsius in August.

All cases with a diagnosis of chilblains presenting to Injibara General Hospital during 2024 were identified prospectively. Twelve cases with a final diagnosis of isolated idiopathic chilblains were included in the final analysis. All were residents in the Injibara town and Tilli town. The data presented detailed findings on history and examination, as well as any prescribed treatments and ultimate outcomes to date.

## Case presentation

**Figure 1:**
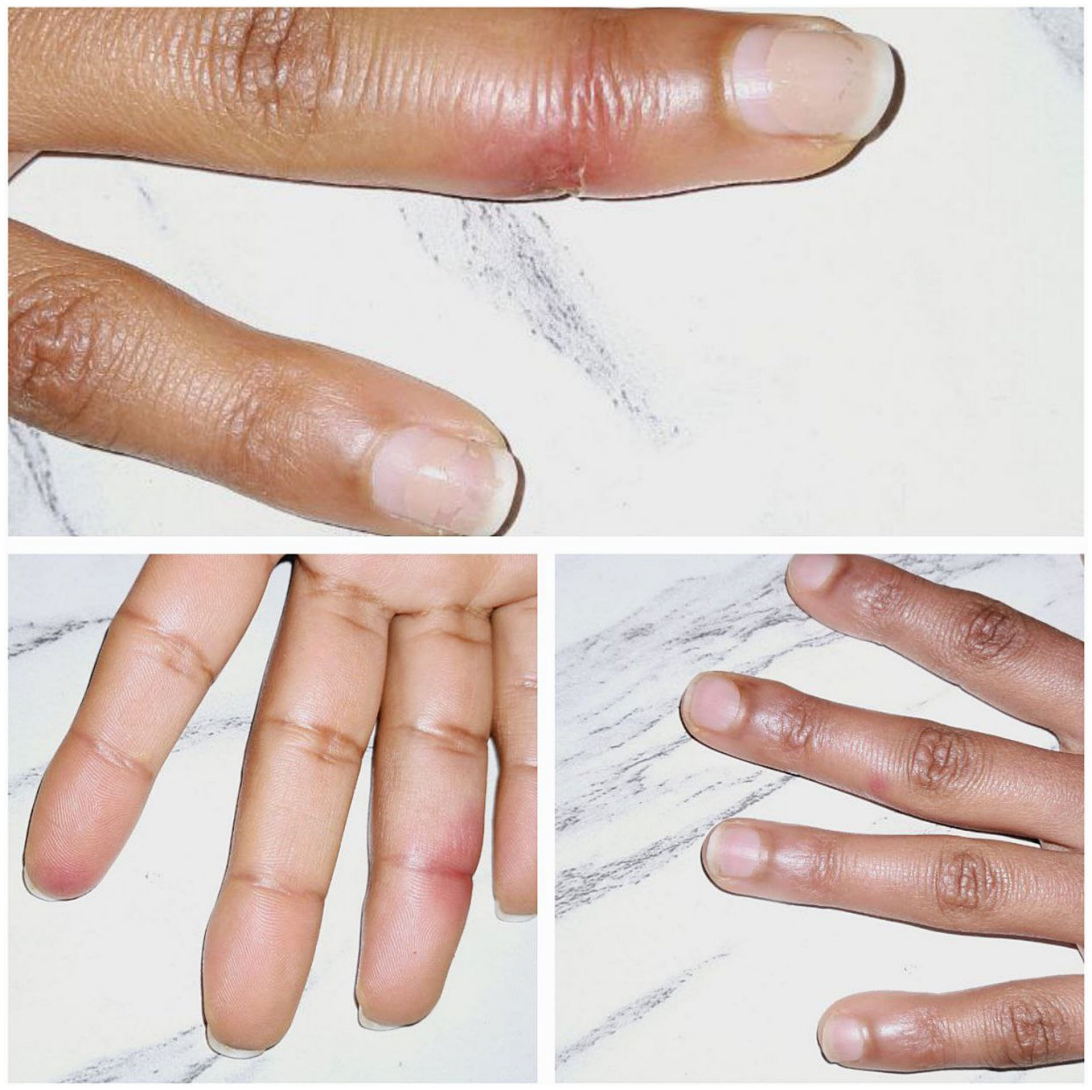
Erythematous macules and plaques with erosions involving the fingers.

**Figure 2:**
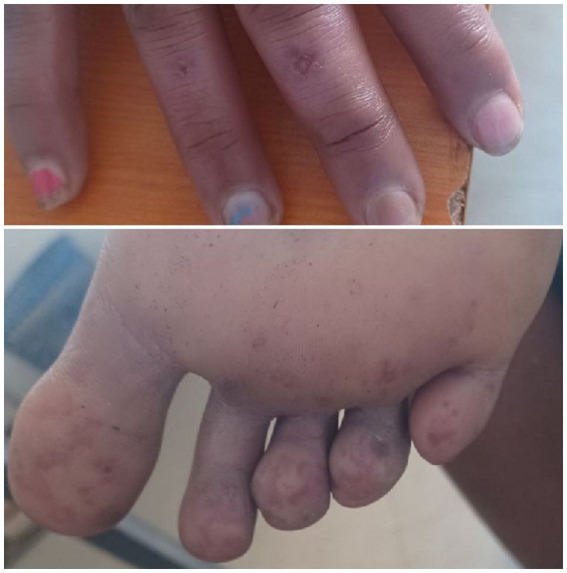
Tender macules and papules affecting fingers and toes bilaterally.

Twelve patients who were diagnosed with idiopathic chilblain in 2024 at Injibara General Hospital in Ethiopia are presented here. Two out of twelve patients in need had already sought therapy elsewhere with unspecified medications before arriving at the hospital but without any improvement in their skin condition. Ten patients arrived early with lesions lasting no more than two weeks. All of our patients came for the relief of symptoms and two were worried for the deformity brought by the disease to their fingers. Based on clinical presentations and laboratory investigation, the clinical diagnosis of every patient was independently agreed upon by two dermatologists. Table 1 provides description of the cases, while Figures 1, 2 display case samples.

**Table 1:** Characteristics of 12 case of idiopathic chilblain (pernio) from the Injibara General Hospital, Ethiopia, 2024.

Characteristics			Value^a^
Average age at diagnosis (years)			22.5
Age range			3–55
Sex		Male	3 (25)
		Female	9 (75)
Body mass index		<18.5	8 (66%)
		18.5–24.9	4 (34%)
Residence		Injibara town	10 (83)
		Tilili town	2 (17)
Affected site		Hands only	6 (50)
		Feet only	1 (8)
		Hands and feet	5 (42)
Symptoms and signs		Pruritus	10 (83)
		Burning/pain	12 (100)
		Tenderness	11 (92)
		Edema	5 (42)
		Ulceration	1 (8.3)
Raynaud phenomenon^b^			2 (17)
Recurrent			1 (8)
Treatment		Conservative measures only	2 (17)
		Topical corticosteroids only	4 (33)
		Topical corticosteroids and nifedipine followed by oral prednisolone	1 (8)
		Topical corticosteroids and nifedipine	5 (42)
Response to treatment	Conservative measures only	Complete response	2/2 (100)
	Topical corticosteroids only	Complete response	4/4 (100)
	Topical corticosteroids and nifedipine followed by oral prednisolone	Partial response	1/1 (100)
	Topical corticosteroids and nifedipine	Complete response	5/5 (90)

a Values are expressed as mean (for age) and as No. (percentage) (for others).

b Defined as intermittent and sequential pallor (white discoloration), cyanosis (blue discoloration), and rubor (red discoloration) of acral sites on cold exposure.

Eleven patients were urban-dwelling female young adults and adolescents. In all patients, lesions were located on the fingers and/or foot. All cases presented with itching sensations and/or pain. Only two patients presented with swelling and color change during cold exposure. One patient presented with ulceration involving the tip of the digits. Otherwise, all patients did not report any family history of similar illness currently or in the past.

Diagnostic tests like complete blood count, viral markers (i.e., hepatitis B surface antigen and hepatitis C antibody test), urinalysis, antinuclear antibody, and erythrocyte sedimentation rate were normal in all tested patients. To rule out related autoimmune diseases, particularly systemic lupus erythematous, we conducted most of the aforementioned tests on young female patients as well as those who presented with joint pain and Raynaud phenomenon.

Five patients were prescribed clobetasol propionate ointment 0.05% (60 gram) daily and oral nifedipine 20 mg three times per day for 2 months. Others were treated with only clobetasol propionate ointment 0.05% daily (i.e., four patients), and two patients refused any medical treatment. All patients were counseled to avoid cold and keep extremities warm. All patients were followed for four months. On average our patients have a minimum of two visits. In their subsequent follow up, only one patient has persistent skin lesions with a slight improvement and the rest completely improved. Regarding the speed of recovery or complete remission, we found no difference between individuals treated with oral nifedipine and those treated with topical corticosteroids. After starting oral prednisolone, a patient with a persistent skin lesion (i.e., a 23 years old female patient) demonstrated a moderate improvement. The patient experienced an outbreak of acne related to prednisolone; ultimately, they stopped taking the drug and went to the holy water (i.e., holy water is water that has been blessed by a religious figure or clergy member). A year ago, the same patient experienced a similar episode, which was resolved after treatment with oral prednisolone. One patient complained headache associated with nifedipine and was given anti-pain and reassured.

## Discussion

This is the first clinical account of Ethiopian cases of idiopathic chilblain. From June to August of 2024, 12 individuals in the dermatology department of Injibara General Hospital in Ethiopia were diagnosed with idiopathic chilblain. Nine (or 75%) of these patients were women. This study’s mean age at diagnosis was 23.2 years (range: 3–55 years), which is younger than the 104 patients’ mean age of 38.3 years from the Mayo Clinic (14). However, a study conducted in Western Australia revealed that the average age was 13.5 years, which is lower than our situation, and the age ranged from 6 months to 17 years (15). According to our research, young adult women are the group most frequently afflicted with idiopathic chilblains, which is in line with the results of other studies (8, 14, 15). The association of chilblains with a low body mass index noted in prior studies (8, 16) also seen with two of our patients, suggests that this might be a predisposing factor for this unusual condition.

In our patients, the most common sites of idiopathic chilblain lesions are the hands and feet, consistent with other study findings (4, 17, 18). Cases were characterized by itching and/or pain over the fingers and toes, which got worsened in the morning and evening with the peak of cold exposure. In our cases, pain was the commonest presentation consistent with study findings from Western Australia (4, 15). Three individuals had reported changes in color and edema following exposure to the cold (4, 15). Compared to the Mayo Clinic study, where 17% of patients experienced concurrent Raynaud symptoms (14), the percentage of patients in this study with Raynaud phenomena was 10%. However, skin ulceration was found in one patient in our case series (18).

Consistent clinical history and physical characteristics support the diagnosis. A complete blood count, antinuclear antibody, serum protein electrophoresis, and viral markers are among the tests used to identify related comorbidities (19). In cases where a diagnosis is unclear, we may utilize histopathology. All of the aforementioned tests were carried out and within the normal reference range, with the exception of serum protein electrophoresis (unavailable) and histopathology (not always necessary) (4). In Table 2, we have outlined the key diagnostic features of chilblains that would be helpful in a clinical setting to make an early and specific diagnosis.

**Table 2:** Diagnostic features of the idiopathic chilblain.

Clinical significance
Present as bilateral erythematous to purplish painful and/or itchy papules, plaques or nodules over the fingers and/or toes Onset during cooler months (June to September) Young, low body mass index, and female sex could potentially be predisposing factors Occasionally associated Raynaud phenomenon Response to conservative measures (warming of affected areas)

The key therapy for chilblains is protecting the affected area and avoiding further cold exposure (4). Although pharmacological modalities like vasodilators have been employed, there are no controlled studies to show if they are effective (4). Randomized trials to confirm the efficacy of local corticosteroid treatment are lacking. But limited data are available supporting the efficacy of nifedipine for chilblain (20, 21). Acute chilblains or pernio is a self-limiting process that usually resolves within days to months; although there can be a chronic form of waxing and waning lesions with persistent cold exposure (4).

It is advised that patients with idiopathic chilblain keep the affected area warm by donning gloves, footwear, and adequately insulated clothes (8, 13, 17, 18). Consequently, we recommended all of our patients avoid cold exposure. We also encourage patients to discontinue smoking, but none of our patients were smokers. Among the most popular pharmacologic treatments are oral nifedipine and topical corticosteroids. Topical corticosteroids were used either alone or in conjunction with nifedipine to treat the majority of our patients. Just one patient received oral prednisolone 30 mg daily for one month. Two patients showed improvements with conservative approaches. Only one patient has persistent disease, and the rest have completely improved, similar to study findings from Prakash and Weisman (4). At their last visit, the majority of our patients saw a considerable reduction in their anxiety. However, it is difficult to know whether the response was due to medication or a change in weather condition. These cases of idiopathic chilblain are likely to present the tip of the iceberg, as they were collected in a single health center within a limited period of 3 months.

Chilblains can cause significant anxiety in patients, requiring clinical diagnosis, anxiety relief, and treatment advice. Anecdotal evidence suggests that dermatologists have encountered idiopathic chilblains comparable to those described in this report from their respective centers in some parts of the country.

## Limitation of the study

This study is limited by inclusion of a very small number of patients, short study period, single center study, and unavailability of some laboratory tests.

## Conclusion and recommendations

This is the first study of its kind to reveal the existence of the disease in the country. According to the current study, young women were the group most frequently impacted by idiopathic chilblain. We propose that the key features of chilblains can be utilized to help distinguish this condition from other entities such as thromboembolic disease or vasculitis.

Idiopathic chilblain in Ethiopia is underreported, necessitating comprehensive studies involving larger patient numbers, longer follow up period, multicenter study, and a focus on prevalence, diagnosis, treatment, and psychosocial impact.

## Data Availability

The original contributions presented in the study are included in the article/supplementary material, further inquiries can be directed to the corresponding author.
